# Pharmacotherapy for Poststroke Cognitive Impairment and Poststroke Cognitive Impairment With Dementia: A Review

**DOI:** 10.1155/srat/6893801

**Published:** 2025-05-18

**Authors:** Renju Ravi, Saibal Das, Tahir Hakami, Prakash B. M., Libby Pushparajan

**Affiliations:** ^1^Department of Clinical Pharmacology, Faculty of Medicine, Jazan University, Jazan, Saudi Arabia; ^2^Indian Council of Medical Research-Centre for Ageing and Mental Health, Kolkata, India; ^3^Department of Global Public Health, Karolinska Institutet, Stockholm, Sweden; ^4^Department of Neurology, St. Gregorios Medical Mission Hospital, Parumala, Kerala, India

**Keywords:** cholinesterase inhibitors, cognitive impairment, pharmacotherapy, poststroke, vascular dementia

## Abstract

Poststroke cognitive impairment (PSCI) refers to any level of cognitive decline occurring after a stroke, ranging from mild to severe impairments, while PSCI with dementia describes a more severe form where the cognitive decline significantly affects daily functioning and meets the clinical criteria for dementia. PSCI occurs in more than half of individuals who have had a stroke. Despite its high prevalence, the pharmacotherapeutic options for PSCI are limited. Several pharmacotherapeutic options like cholinesterase inhibitors (e.g., donepezil, galantamine, and rivastigmine) and *N*-methyl-d-aspartate receptor antagonists (e.g., memantine) have shown potential in improving cognitive functions. However, their overall effectiveness remains inconsistent across different studies and patient populations. Newer drugs such as citicoline, cilostazol, and antidepressants have shown promise, but further research is needed to validate their efficacy and safety specifically for PSCI management.

## 1. Introduction

Stroke is a leading cause of both disability and death worldwide. It is especially prevalent in low- and middle-income countries [1, 2]. Poststroke cognitive impairment (PSCI) is defined as immediate and/or delayed cognitive decline that begins within 6 months after a stroke [3]. Vascular cognitive impairment is a complex spectrum encompassing PSCI and small vessel disease-related cognitive impairment [4]. PSCI is a common occurrence among stroke survivors, occurring in more than half of individuals who have had a stroke. PSCI can affect various cognitive functions, such as attention, memory, learning, and visual–spatial orientation [5]. The prevalence of PSCI may vary depending on the geographic region, the diagnostic criteria used, and the methods of assessment [6].

PSCI refers to any level of cognitive decline occurring after a stroke, ranging from mild to severe impairments. In contrast, dementia following a stroke is a more severe form of cognitive impairment that involves significant, progressive decline affecting daily functioning and meets clinical criteria for dementia. PSCI correlates with demographic aspects like age, education, and occupation, alongside vascular factors and the existence of conditions such as hypertension, dyslipidemia, and diabetes mellitus, as well as differences based on ethnicity and race [7]. While it is often mild in stroke survivors, some studies have found that the prevalence of dementia in poststroke cases can range from 7.4% in a study of first-time strokes to 41.3% in hospital-based cases of recurrent strokes [8]. Previous research has shown that the prevalence of PSCI can vary from 23% to 55% in the first 3 months after a stroke, declining to between 11% and 31% 1 year after the stroke [9, 10]. Several factors are associated with a poor prognosis of PSCI, including severe stroke, advanced age, and multifocal brain damage [11].

PSCI is a complex condition resulting from the interplay between neurovascular damage and neurodegenerative processes, particularly involving Alzheimer's disease (AD). The mechanisms underlying PSCI include direct neuroanatomical damage from stroke, such as injury to the hippocampus, thalamus, and frontal lobes, which disrupts neural networks responsible for cognitive functions like memory, attention, and executive function. Beyond this vascular damage, there is a significant overlap with AD pathology, such as amyloid-beta plaques and tau tangles, which can accelerate cognitive decline poststroke. Stroke can exacerbate underlying AD pathology, while the presence of AD changes can make the brain more susceptible to ischemic injury, creating a vicious cycle of cognitive impairment. This overlap suggests that PSCI mechanisms are not solely vascular but also involve neurodegenerative changes driven by factors like inflammation, oxidative stress, and blood–brain barrier disruption. Genetic factors, such as the APOE *ε*4 allele associated with AD, may also increase the risk of cognitive decline after a stroke, further complicating the interplay between these two conditions. These combined mechanisms highlight the need for therapeutic strategies that address both vascular health and neurodegenerative processes to effectively manage PSCI and prevent its progression to dementia. Future research should focus on elucidating these mechanisms to develop targeted interventions that can modify the course of PSCI.

**Figure 1:**
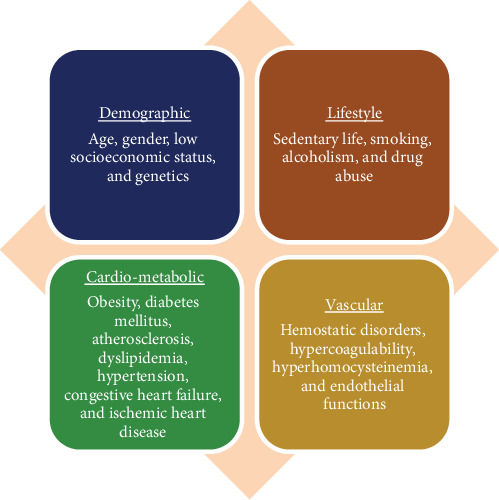
Major risk factors for poststroke cognitive impairment.

The exact mechanisms underlying PSCI are not fully understood. Yet it is thought that various factors could play a role in this condition, encompassing neuroanatomical damage due to stroke in regions like the hippocampus and white matter, cerebral microbleeds stemming from minor cerebrovascular issues, and a convergence of AD and stroke (see Figure 1) [12]. Several measures may help prevent PSCI after a stroke by reducing or controlling vascular disease. In addition, various aspects can increase the risk of PSCI, such as diabetes, hypertension, dyslipidemia, sleep apnea, and obesity [12]. Despite its high prevalence, the pharmacotherapeutic options for PSCI are limited [13]. We herein discuss the role of pharmacotherapy for cognitive improvement after stroke.

## 2. Review Aim and Methods

We aimed to review the literature on the role of pharmacotherapy of PSCI in adults. We searched PubMed, Embase, International Clinical Trials Registry Platform (World Health Organization), Cochrane Library databases (Cochrane Database of Systematic Reviews, Cochrane Central Register of Controlled Trials [CENTRAL], and Cochrane Methodology Register), and two preprint servers (http://medRxiv.org and Research Square) from inception until 31 August 2023. The search terms used in various combinations were “stroke,” “cerebrovascular accident,” “vascular dementia,” “dementia,” “cognitive impairment,” “cognition,” “memory,” “attention,” “speech,” “poststroke cognitive impairment,” “PSCI,” and “cognition enhancer.” The search strings were modified for different bibliographic databases in combination with database-specific filters. Only conventional allopathic drugs were included in this review.

We included studies that specifically focused on PSCI. The inclusion criteria were as follows: (1) studies that investigated PSCI, defined as cognitive decline occurring within 6 months following a stroke and assessed using standardized cognitive assessment tools such as the Mini-Mental State Examination (MMSE), Vascular Dementia Assessment Scale (V-ADAS), and Alzheimer's Disease Assessment Scale-Cognitive Subscale (ADAS-Cog); (2) studies where dementia was diagnosed in the context of stroke, meeting clinical criteria for dementia with significant impairment in at least two cognitive domains affecting daily function; (3) studies that involved human participants only, excluding animal studies and in vitro research; and (4) randomized controlled trials (RCTs), observational studies, and meta-analyses that provided data on pharmacotherapeutic interventions for PSCI. We excluded studies that focused solely on cognitive impairments unrelated to stroke, those that involved mixed or Alzheimer's-type dementias without a clear stroke component, and nonhuman studies.

Following the search, all retrieved studies were screened in two stages: title and abstract screening and full-text review. During the title and abstract screening, studies that clearly did not meet the inclusion criteria were excluded. For the full-text review, studies were assessed based on predefined inclusion criteria: human studies involving adults with PSCI or PSCI with dementia, use of conventional allopathic pharmacotherapies, and provision of data on cognitive outcomes. Data extraction from the included studies was conducted independently by multiple reviewers to minimize bias, focusing on study characteristics, patient populations, interventions, outcome measures, and key findings. Discrepancies were resolved through discussion and consensus. This rigorous process ensured that the review included high-quality evidence pertinent to the pharmacological management of PSCI.

## 3. Review Overall Results

Our review included 50 articles, which comprised a range of study types (observational studies and meta-analyses) to provide a comprehensive overview of pharmacotherapeutic interventions for PSCI. The RCTs included primarily focused on assessing the efficacy and safety of pharmacological agents such as cholinesterase inhibitors (donepezil, galantamine, and rivastigmine), *N*-methyl-d-aspartate (NMDA) receptor antagonists (memantine), antidepressants (nortriptyline and escitalopram), and other miscellaneous drugs (citicoline, nimodipine, cilostazol, propentofylline, naftidrofuryl, vinpocetine, and piracetam) in improving cognitive function in patients with PSCI. These trials varied in sample size, ranging from small pilot studies with fewer than 50 participants to large multicenter studies with over 500 participants. The duration of follow-up in these studies varied from 4 weeks to 24 months, with most studies reporting outcomes at 3–6 months posttreatment initiation. Observational studies provided valuable insights into the real-world effectiveness, tolerability, and safety of these pharmacotherapies. These studies also reported on factors such as medication adherence, side effects, and long-term cognitive outcomes. Meta-analyses included in this review synthesized data from multiple RCTs and observational studies to offer comprehensive evidence on the effectiveness of specific pharmacotherapies for PSCI and PSCI with dementia. They utilized a variety of statistical methods to aggregate data, providing pooled estimates of treatment effects on cognitive outcomes such as the MMSE and ADAS-Cog. Systematic reviews further complemented the findings by summarizing the existing literature and providing an overview of the pharmacotherapeutic landscape for PSCI.

## 4. Pharmacotherapeutic Options for PSCI

The various drugs used for the treatment of PSCI are enumerated in Table 1.

### 4.1. Cholinesterase Inhibitors

Various cholinesterase inhibitors that are approved for clinical use in AD [14] are used for PSCI as follows:

#### 4.1.1. Donepezil

Donepezil is the most promising cholinesterase inhibitor for the treatment of PSCI. The results regarding the effects of donepezil on overall cognitive function in patients with PSCI are inconsistent [15–18]. Four trials have examined the effectiveness of donepezil in PSCI [15–17, 19]. One of these studies, which included almost 1000 patients with probable or possible PSCI, found that taking donepezil led to better performance on a cognitive assessment using V-ADAS score after 6 months compared to a group taking placebo. However, there was no difference between the two groups on another assessment using the Clinicians' Interview-Based Impression of Change plus Caregiver Input (CIBIC-Plus) score. When the researchers looked at a subgroup of patients without brain damage in the hippocampus, they found that cognition improved in the group taking donepezil after 24 weeks, while it remained stable in the placebo group. In patients with damage to the hippocampus, cognition declined in the placebo group but remained stable in the group taking donepezil. No significant or unexpected side effects were seen in the group taking donepezil [17].

In a trial, treatment with donepezil (up to 10 mg daily for 16 weeks) was associated with significant improvements on several clinical tests, including the Western Aphasia Battery, the Communicative Activity Log, and the Psycholinguistic Assessment of Language Processing scores. However, these improvements were not sustained after treatment ended at Week 20, suggesting that the benefits of donepezil may not be related to neural reorganization [20]. In contrast, a double-blind, placebo-controlled, crossover study found that donepezil was ineffective in improving poststroke aphasia and actually negatively affected speech outcomes [21]. Donepezil has been shown to improve oral expression, auditory comprehension, naming, and repetition in some cases [22].

A recent study in patients with stroke affecting the right hemisphere found that treatment with donepezil for 4 weeks was associated with significant cognitive improvement as measured by MMSE [23]. Brain functional magnetic resonance imaging additionally revealed heightened activity in specific regions, such as the prefrontal areas, inferior frontal lobes, and the left inferior parietal lobe. This suggests that the effects of donepezil may be related to the parietofrontal network in the brain, which is involved in cognitive function in patients with PSCI [23]. However, a study of patients with cerebral autosomal dominant arteriopathy with subcortical infarcts and leukoencephalopathy found different results [19]. In this study, the effects of donepezil on 168 such patients were evaluated using the ADAS-Cog score at 18 weeks. The vascular ADAS-Cog scores did not show any notable distinction between the donepezil and control groups. However, the donepezil group did show an improvement in executive function [19]. A meta-analysis included four trials, which included 2361 patients being divided into two groups: one group received donepezil, while the other received placebo [24]. Donepezil was significantly more effective than the placebo in improving the MMSE score, and it showed superior benefits on the ADAS-Cog score compared to the placebo [24]. A study found that treatment with donepezil was associated with significant improvements in cognitive function as measured by the V-ADAS-Cog test in patients with PSCI at 18–24 weeks [25]. Given this evidence, the American Heart Association/American Stroke Association (AHA/ASA) has proposed that donepezil could potentially be effective for improving cognition [25].

#### 4.1.2. Galantamine

Galantamine is approved for treating patients with mild to moderate AD [26, 27]. There is limited information available on the effects of galantamine on PSCI. In a randomized, double-blind trial, 592 patients diagnosed with a diagnosis of probable vascular dementia or AD combined with cerebrovascular disease were given either galantamine (24 mg/day) or a placebo. The results showed that galantamine was more effective than the placebo on the ADAS-Cog and CIBIC-Plus tests. Galantamine was also associated with improvements in activities of daily living as compared to the placebo. However, when the data were analyzed for a subgroup of patients with only PSCI, there were no significant differences between the galantamine and placebo groups on the ADAS-Cog and CIBIC-Plus tests [28]. In another trial, the effectiveness and safety of galantamine in 788 patients with PSCI were evaluated. The results showed that patients treated with galantamine had a greater improvement on the ADAS-Cog test after 26 weeks compared to the placebo group. However, there was no significant difference between the galantamine and placebo groups on the Alzheimer's Disease Cooperative Study–Activities of Daily Living (ADCS-ADL) score at Week 26. The use of galantamine did not significantly improve overall functioning, as measured by the CIBIC-Plus assessment. The group receiving galantamine experienced a greater number of side effects, leading to more participants discontinuing the study due to issues like nausea, diarrhea, vomiting, and falls. Despite demonstrating certain cognitive benefits from galantamine treatment, the study did not show a significant improvement in functional abilities overall [29]. Another trial on the use of galantamine in patients with a combination of vascular dementia and AD found that galantamine treatment was associated with a significantly smaller decline in cognition, function, and behavior compared to the control group [16]. However, a meta-analysis that combined the results of the two trials did not find evidence of the effectiveness of galantamine. The meta-analysis did suggest an increased risk of gastrointestinal adverse effects with galantamine use [26]. The AHA/ASA has issued a statement on the treatment of PSCI. According to this statement, the use of galantamine may be beneficial for patients with a combination of AD and PSCI [25].

For galantamine, while some studies demonstrated its effectiveness in improving cognitive function in patients with vascular dementia or AD with cerebrovascular components, the benefits were not consistently observed in those with isolated PSCI. For instance, in a randomized, double-blind trial involving 592 patients, galantamine showed improvements in cognitive assessments like ADAS-Cog and CIBIC-Plus tests; however, these effects were not significant in the subgroup of patients with only PSCI. This suggests that galantamine may be more effective in mixed pathologies where AD coexists, rather than in cases of pure PSCI, thereby limiting its general applicability. Furthermore, galantamine use was associated with a higher incidence of gastrointestinal side effects, leading to a greater discontinuation rate in some studies.

#### 4.1.3. Rivastigmine

There is limited information available on the effectiveness and safety of rivastigmine for the treatment of PSCI. Some animal research has suggested that rivastigmine may be beneficial for PSCI, potentially by improving blood flow and metabolism in brain tissue damaged due to lack of blood flow and causing blood vessels to relax [30]. In a trial, 710 patients with probable PSCI were randomly assigned to receive either rivastigmine or a placebo and were followed for 24 weeks. Out of the 572 patients who finished the study, 275 belonged to the rivastigmine group. Although the initial assessment using the V-ADAS test displayed a notable gap between the groups, this difference disappeared when considering only those who completed the study [31]. Similarly, no divergence was found in the Alzheimer's Disease Cooperative Study–Clinical Global Impression of Change (ADCS-CGIC) test score. Additional examination revealed that the majority of benefits were evident in older patients (aged 75 years or above), hinting that the observed effect might stem from underlying AD pathology [31]. The observed benefits in older patients could indeed suggest that the efficacy of certain pharmacological treatments, such as cholinesterase inhibitors (e.g., rivastigmine), might be more pronounced in those with coexisting AD pathology. As patients age, the likelihood of mixed dementia where both vascular and AD pathologies coexist increases. In such cases, cognitive decline might not solely be due to cerebrovascular events but also due to the progressive neurodegenerative changes characteristic of AD. This overlap in pathologies may mean that treatments traditionally effective for AD, like cholinesterase inhibitors, show more noticeable effects in this older population.

In another trial, the safety and effectiveness of rivastigmine were evaluated in 50 patients with PSCI without dementia. The patients were randomly assigned to receive either rivastigmine (up to a daily dose of 9 mg) or a placebo for 24 weeks. The study demonstrated that patients in the group taking rivastigmine had a statistically significant improvement on a specific part of a test of verbal fluency compared to those in the placebo group. The rivastigmine group exhibited slight enhancements in the Color Trails Test scores; however, these improvements did not reach statistical significance [32]. Overall, rivastigmine was well tolerated, although certain patients, particularly those with multiple existing health conditions, needed a more gradual dosage increase than outlined in the initial study protocol. Additionally, in a separate study, the impact of rivastigmine treatment (at doses of 3–6 mg/day) was assessed against low-dose aspirin (100 mg/day) in 16 patients diagnosed with subcortical PSCI. The group receiving rivastigmine showed significant improvements in executive function, as measured by the 10-point Clock Drawing Test, and had a reduction in caregiver stress [33]. In contrast, the group receiving low-dose aspirin showed no improvements in any outcome measure and a worsening in clinical dementia rating and executive function. The adverse effects of rivastigmine were generally well tolerated, and long-term use of the drug appeared to be safe. The most common adverse effects were nausea, loss of appetite, low blood pressure when standing up, fainting, and muscle contractions [33]. A study involving 64 patients with probable PSCI followed patients for 16 months and compared the effects of rivastigmine to a combination of aspirin and nimodipine. The results showed that patients in the rivastigmine group had improved executive function, behavior, and daily living activities but did not see improvement in overall cognitive function as measured by the MMSE [34]. The results of the studies on the use of rivastigmine in patients with PSCI suggest that it may have some benefits in improving executive function, but there is little evidence to support its use for improving overall cognitive function or activities of daily living [33, 35]. The AHA/ASA does not currently recommend the use of rivastigmine for this indication [25].

The evidence for rivastigmine's effectiveness in treating PSCI is also inconsistent, with mixed results reported across studies. Some trials indicated potential cognitive benefits, particularly in older patients aged 75 years and above, hinting at underlying Alzheimer's pathology as a potential reason for these observed effects. However, other studies have failed to demonstrate significant improvements in overall cognitive function or activities of daily living when compared to placebo. The inconsistent results, coupled with a lack of robust evidence specifically targeting PSCI rather than mixed dementia cases, make it challenging to draw definitive conclusions about rivastigmine's efficacy in this population. Given these limitations, there is a critical need for more high-quality RCTs that specifically focus on pure PSCI populations, with clear stratification by cognitive domains and rigorous assessment of long-term outcomes. This future research would help in better understanding the therapeutic potential and limitations of these drugs and guide more tailored treatment strategies for patients with PSCI and PSCI with dementia.

### 4.2. Memantine

Glutamate activates the NMDA receptors in the brain. Memantine can protect brain cells from damage and improve thinking and function in people with different types of dementia [36–38]. Memantine is a noncompetitive NMDA receptor antagonist, meaning it works by blocking NMDA receptors rather than activating them as glutamate does. In addition to its effects on NMDA receptors, memantine can reduce brain cell damage and improve cognitive function in both global and focal models of reduced blood flow to the brain, as well as in various stages of dementia [36–38]. Animal studies have suggested that memantine may be effective in improving memory and reducing brain damage caused by cerebral ischemia [39–41]. Another study conducted in rats with cortical occlusions indicated that administering memantine could potentially limit the expansion of microinfarcts and decrease cognitive impairments [42]. In a trial involving 548 patients diagnosed with PSCI, they were randomly divided to receive either memantine or a placebo over 28 weeks. The memantine group displayed substantial enhancements in cognitive function, although there was not a notable shift in overall function as evaluated by the Clinical Global Impression of Change (CGIC) scale by the study's end [43].

In a study involving 321 individuals with mild to moderate PSCI, the efficacy and tolerability of memantine were examined over 28 weeks. Results revealed that memantine exhibited significant improvements in ADAS-Cog test scores, a measure of cognitive function, in comparison to the placebo group. The average score in the memantine group increased by 0.4 points, while the placebo group experienced a decline of 1.6 points, resulting in a 2.0-point difference. However, while the response rate on the CIBIC-Plus assessment for overall function was 60% in the memantine group versus 52% in the placebo group, this disparity did not reach statistical significance. Additionally, the trial determined that memantine was well tolerated without notable adverse effects when compared to the placebo [44]. Two other trials have found that memantine can improve cognitive function as measured by the ADAS-Cog test and behavioral symptoms as measured by the Nurses' Observation Scale for Geriatric Patients (NOSGER) disturbed behavior scale in people with mild to moderate PSCI. However, these trials did not find significant improvements in overall functioning [43, 44]. While the effectiveness of memantine in improving cognitive function after a stroke is not yet clear, animal studies have suggested that it may have the potential for this use [25]. The combination of the two trials in a meta-analysis suggested that while memantine showed positive impacts on cognition and behavior, these effects were not supported by measures assessing overall functioning, such as clinical global measures. This incongruity implies a need for additional research to comprehensively understand the complete effects of memantine on PSCI [36].

### 4.3. Antidepressants

Antidepressants have been studied extensively for their potential role in improving motor function after a stroke [45, 46]. Additionally, researchers have also looked at their potential use in treating PSCI in people with and without poststroke depression. In a RCT evaluating the impact of the antidepressant nortriptyline on cognitive function among individuals with poststroke depression, 47 patients displaying symptoms of minor or major depression, as assessed by the Hamilton Rating Scale for Depression (HAM-D), were included. Notably, only those patients who exhibited an improvement in their mood symptoms also showed enhancements in their cognitive function. This suggests that the effects of antidepressants on cognitive function in people with poststroke depression may be linked to their ability to improve mood [47]. A separate study focused on the effects of the antidepressant escitalopram on cognitive function in people who had had a stroke but did not have depression. The study included 129 patients who were randomly assigned to receive escitalopram, a placebo, or problem-solving therapy. The outcomes of the study indicated a statistically significant enhancement in cognitive function, as evaluated by the Repeatable Battery for the Assessment of Neuropsychological Status (RBANS) total score and the delayed memory score, among the group receiving escitalopram. Interestingly, there were no notable variances in the reported number or types of side effects between the group taking escitalopram and the control group [48].

### 4.4. Miscellaneous Drugs

Several other drugs have been tried with varying results in patients with PSCI. These include the following:

#### 4.4.1. Citicoline

Citicoline (cytidine 5-diphosphocholine) is thought to have neuroprotective effects [49, 50]. A randomized, double-blind trial was conducted to investigate the effects of citicoline in 30 patients with PSCI who were randomly assigned to receive citicoline or placebo twice per day. The results of this trial showed that treatment with citicoline did not lead to any improvements in cognitive function or changes in brain imaging compared to baseline measurements taken at the start of the study and follow-up measurements taken 12 months later [51]. Another open-label trial was conducted to evaluate the safety and potential effectiveness of long-term treatment with citicoline in preventing PSCI among 347 patients who were randomly assigned to receive citicoline or the usual treatment. This study showed that cognitive function improved in all participants at 6 and 12 months after having a stroke. However, compared to the control group, the patients treated with citicoline had better attention-executive functions and temporal orientation outcomes. Quality of life also improved in the citicoline group [52]. In another study, citicoline was found to be effective and well tolerated in patients with mild PSCI [53]. Based on these results, it appears that treatment with citicoline for 12 months in people who have had a stroke caused by ischemic stroke may be safe and potentially effective for preserving certain cognitive functions [54].

#### 4.4.2. Calcium Channel Blockers

Nimodipine has specific effects on small blood vessels and may help to prevent cerebral hypoperfusion, which is a reduction in blood flow to the brain. It is thought to have neuroprotective effects and may help reduce neuronal damage caused by amyloid-beta, a protein associated with AD [55]. In the Scandinavian Multi-Infarct Dementia Trial that analyzed data from a previous trial, patients with PSCI who were given nimodipine performed better on tests of attention and psychomotor performance compared to a group of patients who received a placebo. However, there was no statistically significant improvement in cognitive function for patients with multi-infarct dementia [56]. In another study, patients with cognitive impairment who were treated with nilvadipine showed stable cognitive function for 20 months. In contrast, treatment with amlodipine did not lead to any improvements in cognitive function. Both groups experienced similar reductions in blood pressure, suggesting that the protective effect of nilvadipine on the brain may not be related to its ability to lower blood pressure [57].

#### 4.4.3. Cilostazol

This drug inhibits Type III phosphodiesterase enzyme, which increases cyclic adenosine monophosphate (cAMP) in the body and inhibits platelet aggregation. It also increases cerebral circulation and inhibits lipid peroxidation and apoptosis. It may also reduce the accumulation of amyloid-beta protein in the brain, act as a scavenger of certain harmful chemicals called hydroxyl and peroxyl radicals, and inhibit cell damage. It may also have a protective effect on the white matter due to its anticytotoxic and antiapoptotic effects [58–63]. In a study using rats, the neuroprotective effects of this medication were observed to be related to the activation of a particular pathway in the brain called the cAMP-responsive element-binding protein pathway. This pathway leads to the increased production of two specific proteins: Bcl-2 and cyclooxygenase-2 [62]. In a study using animals, cilostazol was found to have protective, antioxidant, and anti-inflammatory effects when used in combination with simvastatin to treat PSCI [63]. In a small study of 10 patients with both AD and cerebrovascular disease, the addition of cilostazol to donepezil for 6 months resulted in an increase in MMSE scores from baseline [61].

#### 4.4.4. Propentofylline

It is a xanthine derivative that works by blocking the reuptake of adenosine by neurons and glial cells. It also inhibits cAMP and cyclic guanosine monophosphate (cGMP) phosphodiesterase. In animal studies, adenosine has been shown to reduce neuronal cell death and improve brain function in conditions of global and focal ischemia. Additionally, adenosine has been shown to enhance memory and learning in guinea pigs by long-term potentiation in the hippocampus [64]. Four randomized clinical trials have been conducted in a total of 901 patients with mild to moderate AD and 359 patients with mild to moderate PSCI. The results showed that propentofylline (300 mg three times per day for up to 12 months) was significantly more effective than a placebo in improving cognitive and functional outcomes in patients with PSCI, as measured by the Gottfries–Brane–Steen (GBS) Scale and the activities of daily living scale. Propentofylline was well tolerated [65].

#### 4.4.5. Naftidrofuryl

It is an antagonist of a specific type of serotonin receptor (5-HT_2_) receptor. It can reduce oxygen consumption during cerebral ischemia [66]. In a randomized, double-blind trial, there was a significant improvement in cognition with naftidrofuryl in terms of ADAS-Cog and MMSE scores in patients with vascular or mixed dementia [67]. In a trial, the effectiveness of naftidrofuryl was tested in patients with PSCI. The trial was randomized, double blind, and included two groups of patients who received either 400 or 600 mg of naftidrofuryl per day. A control group of patients received a placebo. The study found that significantly more patients in the treatment groups who received naftidrofuryl showed no deterioration on cognitive tests (the ADAS-Cog and SCAG) compared to the placebo group. The benefits of naftidrofuryl were statistically significant at both doses (400 and 600 mg) [66].

#### 4.4.6. Vinpocetine

It is a synthetic derivative of the vinca alkaloid vincamine. In a study of patients with PSCI, vinpocetine 5 mg TID improved cognitive function on ADAS-Cog and MMSE scores [68, 69]. In a randomized, double-blind, placebo-controlled trial of patients with PSCI, vinpocetine improved cognitive function on ADAS-Cog and MMSE scores after 8 weeks of treatment [70]. Another randomized placebo-controlled trial of vinpocetine in PSCI found improvement in cognitive function on the ADAS-Cog after 4 weeks of treatment [71].

#### 4.4.7. Piracetam

Piracetam is a nootropic drug; however, there is insufficient evidence to support its use for the treatment of PSCI. In studies, piracetam has been shown to improve cognition when compared to a placebo, but the studies were not statistically powered. Additionally, piracetam has few serious adverse effects [68]. Piracetam is not recommended as a standard treatment for PSCI based on the currently available evidence [72].

## 5. Newer Drugs for PSCI Under Development

The newer drugs for PSCI that are under Phase 3 clinical trials are akatinol, memantine, butylphthalide, and pimavanserin. The newer drugs for PSCI that are under Phase 1/2 clinical trials are *N*-acetylcysteine, delta-9-tetrahydrocannabinol + paracetamol, and metformin. The newer drug for PSCI that is under Phase 4 clinical trials is paracetamol [73]. The development of newer drugs for PSCI, such as akatinol memantine, butylphthalide, pimavanserin, *N*-acetylcysteine, delta-9-tetrahydrocannabinol combined with paracetamol, and metformin, represents a promising frontier in the management of cognitive deficits following a stroke. These drugs, currently undergoing various phases of clinical trials, are being explored for their potential neuroprotective, anti-inflammatory, and cognitive-enhancing properties. The introduction of these newer pharmacotherapies could significantly change clinical practice by offering more targeted and potentially more effective treatment options for patients with PSCI, especially in cases where traditional drugs like cholinesterase inhibitors and NMDA receptor antagonists have shown inconsistent results. If proven effective, these newer agents could provide clinicians with a broader array of tools to tailor treatments based on the specific pathophysiological profiles of patients, such as those with mixed pathologies involving both vascular and neurodegenerative components.

From a public health perspective, the development and potential approval of these newer drugs could help address the substantial burden that PSCI imposes globally. Stroke survivors with cognitive impairment often face significant challenges in daily functioning, quality of life, and independence, which in turn increases healthcare utilization and economic costs. Effective pharmacological interventions that can slow or reverse cognitive decline could reduce the need for long-term care and rehabilitation services and help maintain the quality of life for stroke survivors. Moreover, these developments underscore the need for an integrated approach to stroke management, combining acute stroke care with long-term strategies to mitigate cognitive decline. Public health initiatives could focus on raising awareness about the importance of early cognitive screening and intervention in stroke survivors, promoting adherence to new treatment protocols, and supporting research efforts to further understand the mechanisms of PSCI. Ultimately, the findings on newer drugs under development for PSCI have the potential to reshape the landscape of stroke recovery, making cognitive rehabilitation a central pillar of poststroke care.

## 6. Discussion

**Figure 2:**
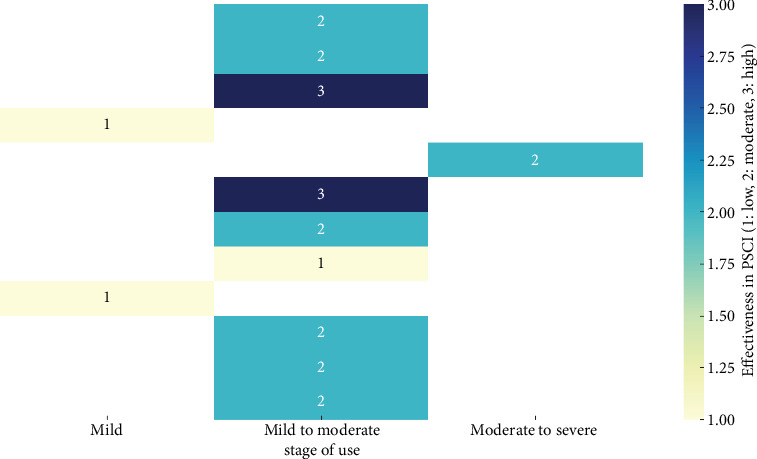
Heatmap representing the effectiveness of various drugs used for poststroke cognitive impairment.

Based on the review of pharmacotherapy for PSCI, it is evident that while several drugs have been explored, the evidence supporting their effectiveness remains varied and context-dependent (Figure 2). Cholinesterase inhibitors, such as donepezil, galantamine, and rivastigmine, have shown some promise in improving cognitive function, particularly in domains like memory and executive function. However, their effectiveness appears to be more consistent in the early stages of PSCI or when dementia is not yet fully developed. Memantine, an NMDA receptor antagonist, also demonstrated potential benefits, particularly in enhancing executive function and managing behavioral symptoms. Despite these findings, the overall efficacy of these pharmacological agents is inconsistent, with some studies showing minimal to no benefit compared to placebo, particularly in later stages of PSCI or in more heterogeneous patient populations. This inconsistency may be due to variations in study design, population characteristics, timing of treatment initiation, and the specific cognitive domains assessed. Overall, the evidence suggests that the timing of treatment initiation and the specific pharmacological agent used can significantly influence the cognitive domains that benefit from therapy. Early initiation, particularly within the first 6 months poststroke, appears crucial for maximizing cognitive recovery, especially in domains such as memory, attention, and executive function.

Additionally, newer and adjunctive treatments, such as citicoline, nimodipine, cilostazol, and antidepressants like escitalopram, present a complex picture. While some studies suggest potential benefits, such as improvements in attention-executive function and memory recall, these effects are not universally observed across all patient populations or study designs. For example, while citicoline and cilostazol have demonstrated some neuroprotective effects in improving cognitive function poststroke, these benefits are often accompanied by increased side effects or require combination therapies for optimal outcomes. The findings suggest that future research should focus on personalized medicine approaches, identifying specific patient characteristics, such as the stage of cognitive impairment, underlying pathologies, and comorbidities, to tailor treatments more effectively. Moreover, there is a need for large-scale, well-designed trials that can provide more definitive evidence regarding the efficacy of these pharmacotherapies, especially in understudied subgroups and for drugs currently in clinical trials. This comprehensive approach could lead to more targeted and effective interventions for managing PSCI and its progression to more severe forms like dementia.

The included studies varied in terms of the stage of PSCI at which treatment was initiated, with most trials commencing treatment within the first 6 months poststroke, a period often associated with the most significant cognitive recovery potential. For instance, several RCTs involving cholinesterase inhibitors, such as donepezil and galantamine, initiated treatment within 3–6 months poststroke and reported cognitive improvements, particularly in patients with mild to moderate PSCI. In contrast, studies on NMDA receptor antagonists like memantine included patients at various stages of PSCI, ranging from mild impairment to more severe forms, demonstrating benefits across different stages but with the most pronounced effects in the early to middle stages of impairment.

The evidence on the efficacy of donepezil and rivastigmine for treating PSCI is inconsistent, with some studies reporting significant cognitive benefits while others show minimal or no improvement compared to placebo. These discrepancies could arise from several factors. First, variations in study design, such as differences in sample size, duration of follow-up, and outcome measures, can significantly impact results. For instance, some trials on donepezil have shown improvements in memory and executive function, particularly in patients without hippocampal damage, while other studies found no significant difference in global cognitive measures. This inconsistency suggests that the effectiveness of donepezil may be influenced by the specific cognitive domains assessed and the degree of underlying brain pathology.

For rivastigmine, the conflicting results may stem from the heterogeneity in patient populations and the presence of mixed pathologies, such as the coexistence of AD pathology with vascular cognitive impairment. Some studies have reported cognitive benefits of rivastigmine primarily in older patients, possibly due to underlying AD pathology, while others failed to show any meaningful impact on overall cognitive function in pure PSCI populations. Additionally, differences in dosing regimens, adherence rates, and the severity of cognitive impairment at baseline could also contribute to these variations in findings. To reconcile these conflicting results, future research should focus on well-defined patient subgroups, standardized outcome measures, and longer follow-up periods to better understand the contexts in which these drugs may or may not be effective for PSCI.

Regarding the specific cognitive domains that showed improvement, several studies indicated differential effects depending on the pharmacological agent used. Memantine, an NMDA receptor antagonist, demonstrated significant benefits in enhancing executive functions, such as decision-making and cognitive flexibility, and in reducing behavioral symptoms, although its effects on memory and language were less pronounced. The use of antidepressants like escitalopram was associated with specific improvements in delayed memory recall and overall cognitive recovery in patients without depression, suggesting its potential benefit in mood-regulated cognitive functions. Other agents, such as citicoline and cilostazol, were reported to improve attention and executive function domains, such as mental processing speed and working memory, while nimodipine was more effective in enhancing psychomotor performance and attention span. By including these specific details about the cognitive domains affected, we provide more comprehensive insights into the benefits and limitations of each pharmacotherapy for managing PSCI and PSCI with dementia, thereby helping clinicians and researchers understand which cognitive functions may benefit the most from particular interventions. This addition aims to give a more nuanced understanding of the impacts of these pharmacotherapies beyond the assessment tools or batteries used in the studies.

While several pharmacotherapies have been explored for the treatment of PSCI, it is important to consider their safety profiles and potential adverse effects. Cholinesterase inhibitors, such as donepezil, galantamine, and rivastigmine, are commonly associated with gastrointestinal side effects like nausea, vomiting, and diarrhea, as well as fatigue, insomnia, and, occasionally, bradycardia or confusion, particularly in elderly patients [74]. Memantine is generally well tolerated but can cause dizziness, headaches, confusion, and agitation [75, 76]. Antidepressants like escitalopram and nortriptyline may lead to dry mouth, drowsiness, dizziness, and an increased risk of falls, with nortriptyline also posing risks of orthostatic hypotension and cardiac arrhythmias [77]. Other drugs, such as citicoline, nimodipine, and cilostazol, have been linked to gastrointestinal discomfort, headaches, hypotension, and palpitations [75, 76]. These adverse effects can impact patient adherence and must be carefully weighed against the cognitive benefits, particularly in vulnerable populations like the elderly and those with comorbidities, emphasizing the need for individualized treatment plans and close monitoring to optimize therapeutic outcomes.

Future research on pharmacotherapies for PSCI should focus on several key areas to address the existing gaps in evidence and improve clinical outcomes. First, there is a need for more large-scale RCTs that specifically target homogeneous populations with pure PSCI, rather than mixed dementia cases. These studies should include clear stratification based on the severity and stage of cognitive impairment (mild, moderate, or severe PSCI) and should evaluate the efficacy of both existing and newer pharmacotherapies, such as akatinol memantine, butylphthalide, and *N*-acetylcysteine, across these stages. Additionally, future trials should focus on more precise cognitive domains (e.g., attention, executive function, memory, and language) rather than global cognitive measures alone, to better understand which specific functions are most amenable to pharmacological intervention.

Moreover, there is a need for more research into the long-term effects and safety profiles of these pharmacotherapies, particularly in diverse populations, including older adults and those with comorbidities such as diabetes, hypertension, and depression, which are common in stroke survivors. Longitudinal cohort studies that follow patients for extended periods could provide valuable insights into the sustained efficacy and potential side effects of treatments over time. Furthermore, future studies should explore combination therapies, integrating pharmacological treatments with nonpharmacological interventions like cognitive rehabilitation, physical exercise, and lifestyle modifications, to assess synergistic effects on cognitive recovery. Another crucial area for future research is the development of biomarkers to predict response to treatment, enabling a more personalized approach to managing PSCI. Finally, there is a need for cost-effectiveness studies to evaluate the economic impact of these treatments on healthcare systems, particularly in low- and middle-income countries where the burden of stroke and cognitive impairment are highest. These research directions will help to optimize therapeutic strategies and improve outcomes for patients with PSCI.

## 7. Conclusion

There has been a significant increase in the number of strokes and deaths caused by stroke from 1990 to 2019. This increase has occurred despite a decrease in age-standardized rates, particularly in those over 70 years old. The highest rates of stroke-related mortality and disability-adjusted life years occurred in low-income countries. Without effective primary prevention strategies, the burden of stroke is likely to continue to increase, particularly in low-income countries [2]. PSCI is a significant public health problem causing a massive toll on the global economy [78]. The treatment of PSCI may include the use of antidementia medications, as well as strategies targeting cerebrovascular diseases. However, the effectiveness of these pharmacotherapies is not yet clear, and further research is needed to determine the most effective treatment approaches [12]. Among the different drugs used for PSCI, cholinesterase inhibitors (donepezil, galantamine, and rivastigmine) show maximum effectiveness. The effectiveness of memantine and some antidepressants was also demonstrated. The supporting role of some other newer drugs for PSCI is also being investigated. There is a need for further research and studies to better understand the effectiveness of various therapy strategies for preventing and treating PSCI. Additionally, in order to more effectively understand and treat PSCI, it is necessary to establish a research network that examines the underlying causes of the condition and investigates potential treatments. This should involve a range of studies, including those that look at clinical, imaging, and pathological factors. It may also be beneficial to start studying interventions at an earlier stage, such as in middle age or earlier. Furthermore, new technologies and more traditional cognitive rehabilitation techniques may help improve outcomes for people with PSCI.

## Figures and Tables

**Table 1:** Various drugs used for the treatment of poststroke cognitive impairment (PSCI) and PSCI with dementia.

**Therapeutic class**	**Drug name**	**Mechanism of action**	**Key clinical evidence**	**Patient type**	**Phase of PSCI**
Cholinesterase inhibitor	Donepezil	Inhibits cholinesterase enzyme	Significant clinical improvements in cognitive function in patients with PSCI; mixed results in PSCI with dementia patients	PSCI, PSCI with dementia	Mild to moderate PSCI; moderate PSCI with dementia
	Galantamine	Inhibits acetylcholinesterase and modulates nicotinic receptors	Effective in vascular dementia and Alzheimer's with cerebrovascular components; no significant improvement in isolated PSCI subgroup	PSCI with dementia	Moderate to severe PSCI with dementia
	Rivastigmine	Inhibits acetylcholinesterase and butyrylcholinesterase	Inconsistent results; benefits mostly observed in older patients, possibly due to underlying Alzheimer's pathology	PSCI with dementia	Mild to moderate PSCI with dementia

NMDA receptor antagonist	Memantine	Blocks NMDA receptors in the brain	Significant improvement in executive function and behavioral symptoms in PSCI; mixed results in overall function	PSCI, PSCI with dementia	Mild to moderate PSCI; moderate PSCI with dementia

Antidepressant	Escitalopram	Selective serotonin reuptake inhibitor	Improved cognitive recovery in PSCI patients without depression; potential mood-regulated cognitive benefits	PSCI	Mild PSCI

Other miscellaneous drugs	Citicoline	Enhances brain acetylcholine synthesis	Improved attention-executive function in mild PSCI; better quality of life outcomes were reported	PSCI	Mild to moderate PSCI
	Nimodipine	Calcium channel blocker	Some improvement in attention and psychomotor performance in vascular dementia; inconclusive results in PSCI	PSCI with dementia, vascular dementia	Mild to moderate PSCI with dementia
	Cilostazol	Inhibits Type III phosphodiesterase enzyme	Improvement in cognitive function and executive function, especially in combination therapies	PSCI, PSCI with dementia	Mild to moderate PSCI; moderate PSCI with dementia

## Data Availability

The datasets generated during the current study are available from the corresponding author upon reasonable request.
